# Green Fabrication Approaches of Lignin Nanoparticles from Different Technical Lignins: A Comparison Study

**DOI:** 10.1002/cssc.202101356

**Published:** 2021-10-04

**Authors:** Patrícia Figueiredo, Maarit H. Lahtinen, Melissa B. Agustin, Danila Morais de Carvalho, Sami‐Pekka Hirvonen, Paavo A. Penttilä, Kirsi S. Mikkonen

**Affiliations:** ^1^ Department of Food and Nutrition Faculty of Agriculture and Forestry University of Helsinki P.O. Box 66 00014 Helsinki Finland; ^2^ Department of Chemistry Faculty of Science University of Helsinki P.O. Box 55 00014 Helsinki Finland; ^3^ Department of Bioproducts and Biosystems Aalto University P.O. Box 16300 00076 Aalto Finland; ^4^ Helsinki Institute of Sustainability Science (HELSUS) University of Helsinki P.O. Box 65 00014 Helsinki Finland

**Keywords:** kraft lignin, birch lignin, grass lignin, lignin nanoparticles, green synthesis

## Abstract

The production of lignin nanoparticles (LNPs) has emerged as a way to overcome the highly variable and complex molecular structure of lignin. It can offer morphological control of the lignin polymer, allowing the formation of stable LNP dispersions in aqueous media, while increasing the potential of lignin for high‐value applications. However, the polydispersity and morphology of LNPs varies depending on the lignin grade and preparation method, and a systematic comparison using different technical lignins is lacking. In this study, it was attempted to find a green fabrication method with a distinct solvent fractionation of lignin to prepare LNPs using three different technical lignins as starting polymers: BLN birch lignin (hardwood, BB), alkali Protobind 1000 (grass, PB), and kraft LignoBoost (softwood, LB). For that, three anti‐solvent precipitation approaches to prepare LNPs were systematically compared: 70 % aqueous ethanol, acetone/water (3 : 1) and NaOH as the lignin solvent, and water/aqueous HCl as the anti‐solvent. Among all these methods, the acetone/water (3 : 1) approach allowed production of homogeneous and monodisperse LNPs with a negative surface charge and also spherical and smooth surfaces. Overall, the results revealed that the acetone/water (3 : 1) method was the most effective approach tested to obtain homogenous, small, and spherical LNPs from the three technical lignins. These LNPs exhibited an improved stability at different ionic strengths and a wider pH range compared to the other preparation methods, which can greatly increase their application in many fields, such as pharmaceutical and food sciences.

## Graphical abstract

**Choose your path**: A green fabrication route is discovered to prepare homogeneous and monodisperse lignin nanoparticles with spherical and smooth surfaces, using three different technical lignins as starting polymers. Results show that the acetone/water (3 : 1) method is the most effective approach to obtain such lignin nanoparticles, which can greatly increase their application in many fields (e. g., pharmaceutical and food sciences).

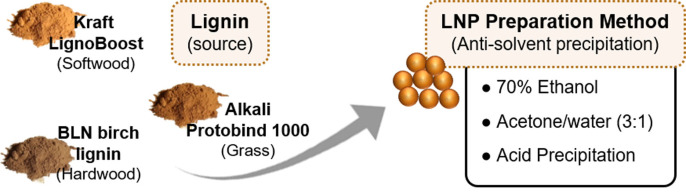

## Introduction

Lignin is a complex polyphenolic macromolecule that is part of the lignocellulosic biomass, and represents one of the most abundant aromatic biopolymers found on Earth.^[1]^ It is primarily composed of three basic monomeric units, *p*‐hydroxyphenyl (H), guaiacyl (G), and syringyl (S), which are interconnected by β‐O‐4 linkages, among others.[^1^, ^2^, ^3^] The proportion of both monomeric units and the type of linkages in the lignin structure can vary according to the biomass source, that is, whether the lignin is isolated from softwoods, hardwoods, or grasses, which ultimately affect the mechanical and physicochemical properties of the lignin polymer.[^4^, ^5^] Despite the high abundance, only around 2 % of the annually extracted lignin has been utilized, mainly as dispersants, additives, and adhesives, while most of the lignin is directly combusted to generate heat and electricity.[^6^, ^7^] During the past years, lignin has gained increased attention from the research community and has shown tremendous potential for advanced applications due to its unique features, such as antioxidant and antimicrobial properties, UV‐blocking ability, biodegradability, and biocompatibility.[^5^, ^8^] However, the lignin valorization has been hampered by its complex and heterogeneous molecular structure, which is highly dependent on the source and extraction method.^[9]^ One way to overcome these limitations is to transform raw lignin into lignin nanoparticles (LNPs), enabling the typically water‐insoluble lignin to form stable colloidal dispersions in water,^[10]^ and increasing the antioxidant activity due to the higher specific surface area.^[5]^ Furthermore, the LNP surface can be easily chemically modified due to the large availability of different functional groups, including aliphatic and phenolic hydroxy and carboxy groups.[^7^, ^11^] Therefore, the development of LNPs allowed them to be employed in several high value‐added applications, including drug delivery,[^12^, ^13^, ^14^, ^15^] antibacterials,[^16^, ^17^] and emulsion stabilizers,[^18^, ^19^, ^20^] among others.

Contrary to the raw lignin powders that are particles with irregular shape and size, the production of LNPs offers a morphological control of the lignin structure, with tunable particle size and shape. For that, several methodologies have been proposed to produce LNPs from different lignin sources, including anti‐solvent precipitation/solvent exchange, interfacial crosslinking, ultrasonication, acid precipitation, and polymerization, as summarized in several reviews.[^5^, ^21^, ^22^] Among these, the most common approach to produce regular spherical LNPs is the anti‐solvent precipitation, where a variety of non‐aqueous solvents (e. g., tetrahydrofuran, dimethyl sulfoxide, ethanol, acetone) can be used to dissolve the raw lignin, and water is used as the anti‐solvent.[^23^, ^24^, ^25^, ^26^, ^27^] The spherical LNPs prepared with this method usually present uniform size, smooth surfaces, and regular shape, which is crucial to achieve high colloidal stability from the point of view of agglomeration and packing.^[22]^ However, the homogeneity and morphology of LNPs prepared with the anti‐solvent precipitation approach varies depending on the lignin grade, as they present different properties (e. g., molar mass, phenolic hydroxy groups, solubility, and purity),[^5^, ^28^] and a systematic comparison is lacking to evaluate those. In this study, we aimed to find a simple, cost‐effective, scalable, and eco‐friendly approach to prepare LNPs using three different technical lignins as starting polymers: hardwood birch lignin (BLN process), wheat straw/Sarkanda grass Protobind™ 1000 (alkali), and softwood LignoBoost (kraft). For that, we systematically compared two anti‐solvent precipitation approaches, where 70 % ethanol or acetone/water (3 : 1) were used as solvents to dissolve the lignin and water as the anti‐solvent, with an acid precipitation approach where the lignin was dissolved in aqueous NaOH solution and precipitated with aqueous HCl. The resulting nanostructures were physicochemically characterized in order to evaluate the effect of the solvent on lignin fractionation during the fabrication of LNPs. The phenolic content on the lignins/LNPs was also quantified using UV spectroscopy and compared with the conventional ^31^P nuclear magnetic resonance (^31^P NMR) spectroscopy. Additionally, the stability of LNPs at different pH (3–8) and ionic strength (10–250 mm) was evaluated in order to select the best approach/solvent to achieve stable, spherical, and homogeneous LNPs from the three technical lignins.

## Results and Discussion

### Influence of the preparation method on the LNP yield

**Scheme 1:**
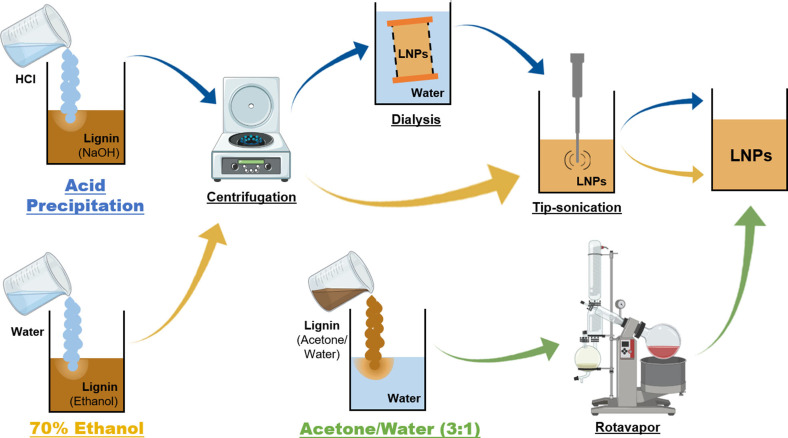
Overview of the different approaches for the LNP preparation used in this study: acid precipitation (blue arrows), 70 % ethanol (yellow arrows), and acetone/water 3 : 1 (green arrows).

The anti‐solvent precipitation is the most common approach to achieve spherical LNPs, with uniform size and smooth surfaces, which enable the application of these LNPs for different areas, including drug delivery and emulsion stabilizers.[^5^, ^22^] However, a common method to achieve such LNPs using non‐harsh solvents from different technical lignins is missing, mainly because the successful preparation of homogeneous and spherical LNPs using the anti‐solvent precipitation approaches depends on the lignin source and its extraction process, which can affect the composition and properties of the raw lignin, such as solubility and amount of phenolic hydroxy groups.[^5^, ^28^] Therefore, we used three different technical lignins as starting polymers for the preparation of LNPs: softwood kraft LignoBoost (LB), wheat straw/Sarkanda grass alkali Protobind™ 1000 (PB), and hardwood BLN birch lignin (BB), rendering LB‐LNPs, PB‐LNPs, and BB‐LNPs, respectively. In order to find a simple method to prepare LNPs from these raw lignins, we used three eco‐friendly anti‐solvent precipitation approaches, where 70 % ethanol, acetone/water (3 : 1), and aqueous NaOH solution were used as the solvent to dissolve the lignin, and water/aqueous HCl as the anti‐solvent, followed by the procedures summarized in Scheme 1. The different solvent/anti‐solvent treatments applied to produce the LNPs will fractionate the three technical lignins in a way that only a fraction of the original lignin will precipitate. In this study, we aim to understand how this solvent fractionation of lignins will ultimately affect the LNP properties.

Regarding the acid precipitation approach, the optimization of the precipitation conditions was done using different acids (HNO_3_, H_2_SO_4_, and HCl), as previously reported by Agustin et al.^[18]^ Here, we selected HCl because it rendered the highest LNP yield among the acids.^[18]^ Therefore, the three raw lignins were dissolved with an aqueous NaOH solution, precipitated with aqueous HCl, and further submitted to centrifugation, dialysis to get rid of salts, and ultrasonication to redisperse the LNPs. When considering the amount of LNPs obtained after precipitation with respect to the initial amount of raw lignin, the percentage yield of LB‐LNPs, PB‐LNPs, and BB‐LNPs using the acid precipitation approach was 85, 81, and 93 %, respectively. In the ethanol method, initially used by Sipponen et al.,^[13]^ the raw lignins were dissolved with a 70 % ethanol solution at the initial lignin concentration of 10 mg mL^−1^, which was found to produce small LNPs while keeping a relatively high LNP yield after optimization of the conditions. After precipitation of the lignin solutions with water until the amount of ethanol dropped to 25 %. The mixtures were then stirred overnight in order to evaporate residual ethanol, and then the LNPs were collected by centrifugation and redispersed using ultrasonication. With this methodology, the percentage yield of LB‐LNPs, PB‐LNPs, and BB‐LNPs decreased to 46, 53, and 53 %, respectively, compared to the acid precipitation method. Finally, the acetone/water approach started with the dissolution of the raw lignins with an acetone/water (3 : 1) mixture at the initial lignin concentration of 10 mg mL^−1^ that was added to the water under vigorous stirring, and the acetone was further evaporated using rotavapor. This method represents a simpler approach to prepare LNPs than the previously used methodologies, with a high percentage yield of about 86 % for all studied LNPs.

The difference in the LNP yields can be affected by the different dissolution efficacy of lignins that vary with their molar mass, as the solubility of lignin increases and the phenolic hydroxy content increases with the decrease in the molar mass.^[29]^ From the technical lignins used here, the LB presents the highest molar mass compared to PB and BB, as determined by size exclusion chromatography (SEC) (Table S1). As a result, the solubility of LB was lower than the PB and BB in the different solvents, and consequently, the LB‐LNPs yields were smaller than PB‐ and BB‐LNPs. The LNP yield could also be affected by the different solubility of raw lignins according to the type and polarity of the solvent used in the LNP preparation.^[29]^ The solubility of lignin seems to be higher in the presence of solvents of intermediate polarity, including ethanol and acetone. Moreover, the addition of up to 30 % of water to such organic solvents has shown a better solubility of the lignin polymer than the individual solvents, which can be due to the formation of hydrogen bonds between hydrates and lignin, leading to an improved lignin dissolution.[^13^, ^29^, ^30^] Additionally, the use of ethanol or acetone as solvent for lignin dissolution in anti‐solvent precipitation approaches compared to the harsh and widely used tetrahydrofuran (THF) is advantageous due to their eco‐friendly properties. Furthermore, it allows to increase the initial concentration of raw lignin used for the LNP preparation without increasing the final size and compromising the polydispersity index (PDI) of the LNPs, enabling a larger‐scale production of LNPs.^[25]^

### Effect of the preparation method on the size and morphology of the LNPs

**Figure 1:**
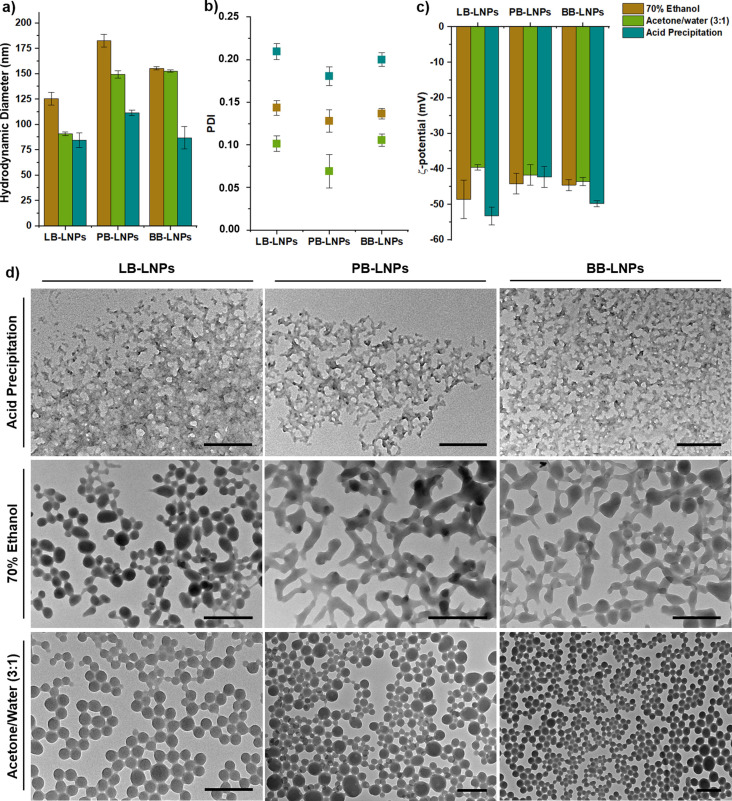
Characterization of LB, BB, and PB‐LNPs by dynamic light scattering in terms of (a) size, (b) polydispersity index (PDI), and (c) ζ‐potential. (d) TEM images of the LB, BB, and PB‐LNPs synthesized using the acid precipitation, 70 % ethanol, and acetone/water (3 : 1) approaches. Scale bars are 200 nm. Error bars represent the mean±s.d. (n≥3).

The as‐prepared LNPs were further characterized for their physicochemical characteristics by determining hydrodynamic diameter, PDI, and ζ‐potential of the LNPs using dynamic light scattering (DLS) (Figure 1a–c), as well as their morphology through transmission electron microscopy (TEM) (Figure 1d).

Non‐covalent forces, such as hydrogen bonding, hydrophobic, and π–π interactions, are known to drive the self‐assembly process of lignin during the particle preparation and can be affected by the nature of the solvent used and the source of lignin.^[31]^ Regarding the nature of the lignin solvent, our DLS results showed that both 70 % ethanol and acetone/water (3 : 1) approaches yielded larger LNPs than the acid precipitation approach (Figure 1a). In such organic solvent‐water binary mixtures, the hydrophilic groups of lignin interact with water and the hydrophobic skeleton with the organic solvent during lignin dissolution. Then, when the ratio of water to organic solvent becomes substantially high, lignin tends to self‐assemble into spherical particles.^[32]^ However, the acidification of an alkaline lignin solution leads to precipitation that continues to an irregular network structure and possible sedimentation, as the acidification protonates the charged groups on lignin.^[22]^ These trends in the LNP morphology were also confirmed using TEM (Figure 1d), in which the LNPs prepared with acid precipitation method appear to be like clusters or aggregates of very small particles, while the 70 % ethanol and acetone/water (3 : 1) led to the formation of quasi‐spherical or spherical LNPs, respectively. As for the source of lignin, our results indicated that the LB‐LNPs exhibited slightly smaller particle sizes compared to the PB‐LNPs and BB‐LNPs. This can be attributed to the different molar mass of the technical lignins, which can influence the formation pattern of spherical particles. Usually, the self‐assembling process starts with the precipitation of large‐molecular‐weight molecules (low water‐solubility) that act as nuclei for the growing particles, and then the low‐molecular‐weight fragments of lignin (rich in hydrophilic functional groups) are the last to precipitate and coat the particle surface.^[22]^ An increased molar mass of the lignin led to the production of smaller particles, which can be due to the increased hydrophobic interactions.[^22^, ^33^] As determined by SEC (Table S1), the molar mass of the technical lignins used in our study (LB>BB>PB) seemed to be inversely proportional to the hydrodynamic diameter of the LNPs (LB‐LNPs<BB‐LNPs<PB‐LNPs). Furthermore, the higher amount of aliphatic hydroxy groups on the softwood LB compared to the hardwood BB lignin (Table S2) can also result in the formation of smaller LNPs, due to the reduced intermolecular bonding between lignin molecules. The stronger non‐covalent π‐π interaction between guaiacyl units on LB structure than the interaction between the syringyl units on both PB and BB lignins can also lead to a denser packing of lignin molecules during LNP formation, and therefore, to the smaller average size of LB‐LNPs compared to the PB‐ and BB‐LNPs.^[34]^ According to DLS, all methods allowed the production of homogeneous and monodisperse LNPs, in particular the acetone/water (3 : 1) method, where the LNPs presented PDI values equal to 0.1 or below (Figure 1b). Similarly to other solvent‐exchange approaches, all the LNPs were negatively charged, exhibiting ζ‐potential values lower than −40 mV (Figure 1c). The negative surface charge can be ascribed to the highly abundant phenolic and aliphatic hydroxy and carboxylic groups on the LNP surface, which can lead to the stabilization of the LNPs in colloidal dispersion due to the electric double‐layer repulsion.

**Figure 2:**
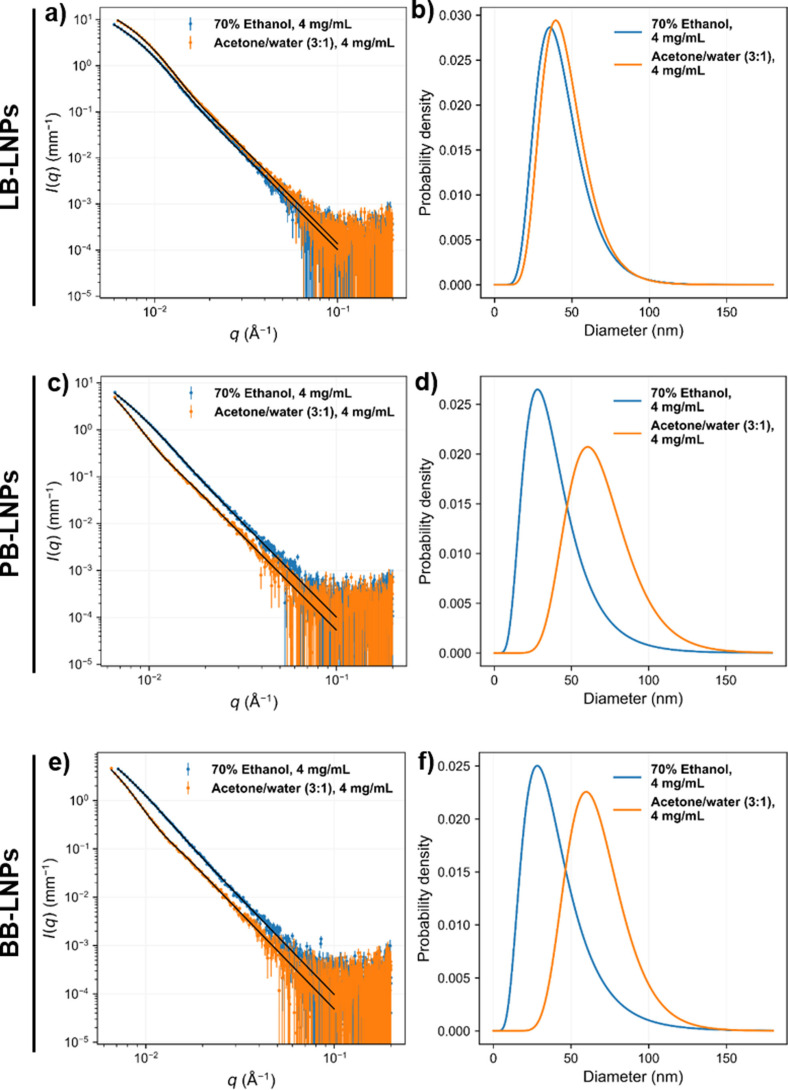
SAXS intensities of LNPs in aqueous solution (points) and fits (solid line) of a model with solid spheres for (a) LB‐LNPs, (c) PB‐LNPs, and (e) BB‐LNPs. The log‐normal distributions of particle diameter resulting from the SAXS fits: (b) LB‐LNPs, (d) PB‐LNPs, and (f) BB‐LNPs.

Small‐angle X‐ray scattering (SAXS) was used to determine the outer dimensions and inner structure of the LNPs in aqueous dispersion at a concentration of 4 or 20 mg mL^−1^. The SAXS intensities of all LNPs prepared with acetone/water (3 : 1) or 70 % ethanol approaches indicated a leveling‐off towards lower values of *q*, which would be expected based on previous synchrotron‐SAXS results from similar LNPs.^[36]^ At higher *q* values, the intensities followed a power law with exponent close to −4, which is characteristic of homogeneous particles with smooth interfaces.^[35]^ The intensities of these samples were fitted with a function corresponding to homogeneous spheres having a log‐normal size distribution with mean radius *R*_mean_ and polydispersity *σ*, following Sipponen et al.^[36]^ The results, converted to particle diameter, are shown in Table S3 and the SAXS fits with the resulting size distributions (assuming log‐normal shape) in Figure 2. Generally, the acetone/water (3 : 1) approach produced larger LNPs than the 70 % ethanol method, except for the LB‐LNPs that presented similar particle size. However, the LNPs fabricated using the acid precipitation approach seemed like aggregates/clusters of smaller particles, rather than large homogeneous particles as observed for the acetone/water and 70 % ethanol fabrication methods. In fact, the acid precipitation approach showed a similar two‐level hierarchical structure formed by LNP aggregation, as reported previously by Agustin et al.^[18]^ Therefore, SAXS intensities of the LNPs prepared via acid precipitation were fitted using the unified exponential/power‐law model.[^18^, ^37^] For comparison between the two fitting models, the SAXS intensities of the acetone and ethanol‐extracted LNPs were also fitted with the unified model but only with one level of structural hierarchy, as represented in Figure S1 and Table S4. The particle diameter determined from the unified fit was larger than the mean diameter calculated using the sphere model, because the sphere model takes into account the polydispersity assuming a log‐normal size distribution, while the unified model assumes monodispersity. When comparing the unified fits of all samples, the overall particle diameter of the LNPs prepared by acid precipitation was generally smaller than that of the LNPs prepared by the acetone/water or 70 % ethanol approaches. Our results also suggested that the particle size measured by DLS is generally larger than the one obtained with SAXS, which can be explained by different factors: (i) the DLS is more sensitive to the presence of larger particles or aggregates than SAXS, and (ii) the complex shape and roughness of the particles and their interaction can also enlarge the hydrodynamic shell, and consequently, the final DLS values.[^38^, ^39^] Nevertheless, SAXS results were found to be more similar to the ones observed using TEM.

### Effect of the lignin fractionation on its chemical structure

**Figure 3:**
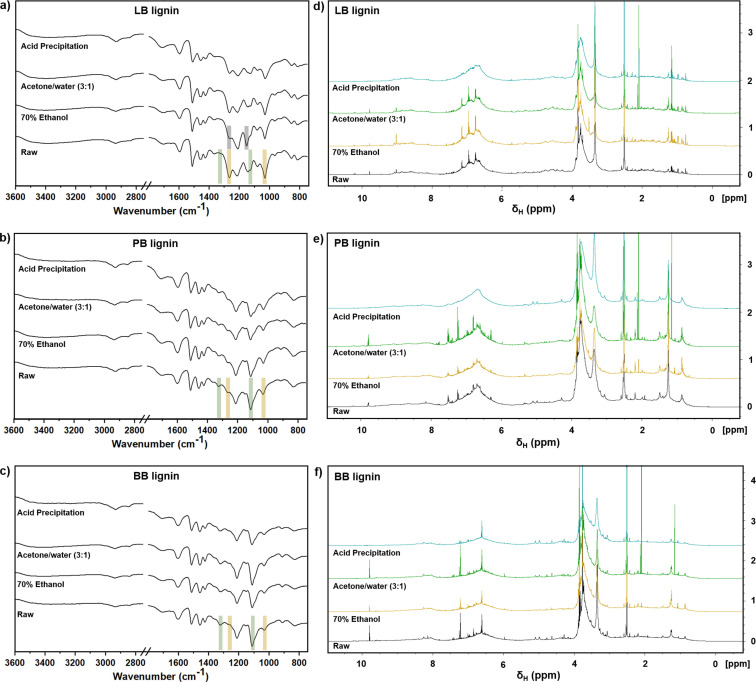
(a–c) FTIR spectra and (d–f) ^1^H NMR of raw LB, PB, and BB lignins and corresponding LNPs.

In order to understand the effect of the solvent on the lignin fractionation during the LNP preparation, the chemical characteristics of raw lignin and respective LNPs were analyzed and compared using Fourier‐transform infrared (FTIR) spectroscopy, proton nuclear magnetic resonance (^1^H NMR) spectroscopy, and the two‐dimensional (2D) heteronuclear single‐quantum coherence (HSQC) 2D NMR spectra. Regarding the FTIR spectra (Figure 3a–c), all the raw lignins and derived LNPs presented the typical infrared bands ascribed to the presence the different functional groups, including phenol hydroxy groups (3500–3100 cm^−1^), carbonyl groups (1600 cm^−1^), and aromatic skeletal bands (1427–1512 cm^−1^), which are summarized in Table S5. However, although both PB and BB raw lignins presented very similar spectra, some featuring differences compared to the LB raw lignin can be observed regarding their compositions on syringyl and guaiacyl units. In the region between 1000 and 1300 cm^−1^, the LB lignin exhibited increased bands at around 1030 and 1270 cm^−1^ ascribed to different vibrations in the aromatic guaiacyl rings (Figure 3a), while the PB and BB raw lignins (Figure 3b,c) presented more intensive bands at 1130 and 1330 cm^−1^ attributed to the aromatic syringyl rings.[^4^, ^40^] These differences are related to the intrinsic structure of the technical lignins derived from different sources, that is, the softwood lignin presents a higher amount of guaiacyl units, while the hardwood lignin contains more syringyl units.[^4^, ^5^] These observations were also confirmed by ^31^P NMR spectroscopy, which indicated that the S/G ratio was 0.87 and 4.27 for softwood LB and hardwood BB, respectively (Table S2). Comparing the FTIR spectra of the raw lignins with the respective LNPs, no substantial differences were observed, with the exception of the LB‐LNPs prepared with the 70 % ethanol method (Figure 3a), which can indicate some structural changes after fractionation of the LB raw lignin during the preparation of LNPs.

^1^H NMR spectroscopy was also conducted to evaluate the structural changes after the lignin fractionation driven by the different approaches to prepare LNPs (Figures 3d–f). The three types of lignin present the typical signals ascribed to the aliphatic (2.25–0.00 and 6.00–4.05 ppm), methoxy (4.05–3.45 ppm), aromatic rings (8.00–6.00 ppm), and phenolic groups (9.35–8.00 ppm).^[41]^ The three lignins exhibited signals related to aldehyde (9.7 ppm) and methoxy (3.8 ppm) groups.[^4^, ^42^] The LB lignin showed peaks at 6.7 and 6.9 ppm that can be attributed to the G2 and G6 of the guaiacyl units, respectively, which are highly abundant in softwood lignin (Figure 3d), while the BB lignin presented an intense peak at 6.6 related to the syringyl units (Figure 3f).^[43]^ Additionally, the PB lignin presented a broad signal around 7.3–7.6 ppm that is attributed to the *p*‐hydroxyphenyl units present in grass lignins (Figure 3e).[^4^, ^44^] When looking into the effect of the lignin fractionation during the LNP preparation, the acid precipitation method showed decreased intensity in the signals attributed to the syringyl and guaiacyl units in the range between 6.5 and 7.5 ppm, as well as for the methoxy group at 3.8 ppm, for the three types of lignin. Overall, the acetone method was revealed to be the best approach to fabricate LNPs, because it presents the most similar signals to the raw starting lignin.

### 2D HSQC NMR analysis

**Figure 4:**
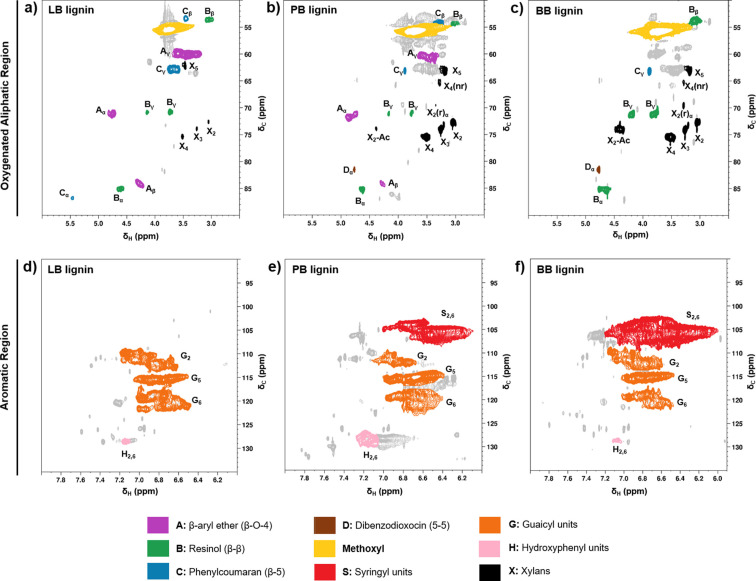
Oxygenated aliphatic and aromatic regions in the 2D HSQC NMR spectra of lignins: (a, d) softwood kraft lignoboost (LB); (b, e) wheat straw soda Protobind 1000 (PB); (c, f) hardwood BLN birch lignin (BB).

The 2D HSQC NMR experiments were conducted to elucidate the chemical structure of the raw softwood LB, wheat straw PB, and hardwood BB raw lignins with an excellent resolution and sensitivity (Figure 4). The spectra of all samples were identical to the previously reported HSQC spectra of these technical lignins, and the main functional groups and linkages on the LB, PB, and BB lignin structures are summarized in Tables S6. In addition to the lignin structures, the relevant signals for polysaccharides linkages are also overviewed in Table S7. Here, we focused our analysis in two main regions of interest: (i) aliphatic oxygenated side chain region (*δ*_C_/*δ*_H_=50–90/2.5–5.8 ppm), and (ii) aromatic/unsaturated region (*δ*_C_/*δ*_H_=90–150/5–8.5 ppm).

In the oxygenated aliphatic region of the 2D HSQC NMR spectra, the methoxy group at *δ*_C_/*δ*_H_=55.5/3.75–3.82 ppm was present in the three types of lignins (Figure 4a–c). The known aliphatic inter‐unit linkages β‐O‐4 (β‐aryl ether α, β, and γ), β‐β (resinol α, β, and γ), and β‐5 (phenylcoumaran α, β, and γ) were identified for the technical lignins.[^45^, ^46^] Regarding the aromatic region of LB lignin (Figure 4d), the main signals correspond to the guaiacyl units that are largely present in softwood lignins: G2 (*δ*_C_/*δ*_H_=110.7–112.4/6.9 ppm), G5 (*δ*_C_/*δ*_H_=115.5/6.9 ppm), and G6 (*δ*_C_/*δ*_H_=120/6.8 ppm).[^45^, ^46^] Besides the signals ascribed to the guaiacyl units, the PB (Figure 4e) and BB lignins (Figure 4f) also showed the characteristic signal ascribed to the syringyl units (C_2,6_–H_2,6_ correlation) at *δ*_C_/*δ*_H_=103.6/6.68. Moreover, the signal for the C_2,6_–H_2,6_ associated to the *p*‐hydroxyphenyl units was also detected at *δ*_C_/*δ*_H_=127.5/7.21 ppm.^[47]^

In addition to the lignin structures, several signals originated from carbohydrate structures were detected in the three technical lignins (Table S7), with particular emphasis to the relatively high xylan contents (Figure 4 a–c).

### Molar mass distribution

**Figure 5:**
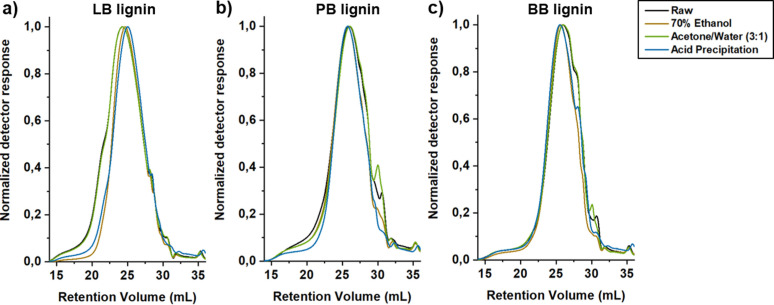
SEC chromatograms of all the raw lignins and respective LNPs: (a) softwood kraft LignoBoost (LB); (b) wheat straw/Sarkanda grass alkali Protobind 1000 (PB); (c) hardwood BLN birch lignin (BB).

The molar mass distribution of the different raw lignins and respective LNPs was determined by SEC. Several reports have discussed some difficulties associated with the molar mass determination of lignin samples using SEC, since there are no proper lignin standards and the determination of molar mass is strongly dependent on the calibration method and experimental set‐up used (e. g., eluent and type of columns).[^4^, ^48^] Therefore, we used identical experimental conditions to study the effect of the solvent used for LNP preparation on the molar mass distribution, and we compared the SEC chromatograms of the LNPs with the raw lignin (Figure 5). The use of solvents with different polarity drives lignin fractionation during the LNP preparation, yielding LNPs with different weight‐average molecular weight (*M*_w_), as shown in Table S1. This can be ascribed to the ability of the solvent to interact with lignin fragments presenting different sizes, amounts and types of functional groups.^[49]^ Generally, the highest polarity solvent used to solubilize lignin before the anti‐solvent precipitation (i. e., ethanol) produced LNPs with the lowest *M*_w_ values, which can be due to the amphiphilic character of ethanol that dissolve a narrow range of low molecular weight fragments. However, when a lower polarity solvent (acetone) was used to dissolve the lignin, the resulting LNPs exhibited higher *M*_w_ values, suggesting that acetone can solubilize a wider range of lignin fragments. Additionally, the SEC chromatograms of the LNPs prepared using the acetone/water (3 : 1) approach were also more similar to the raw lignin, confirming the ability of acetone to solubilize a broader range of lignin fragments (Figure 5). Furthermore, the high‐molecular‐weight fragments of LB lignin (Figure 5a) were not recovered during the acid precipitation or 70 % ethanol approaches, probably because these fragments could not be solubilized in 70 % ethanol and were further removed during the filtration step before the precipitation in water, or then the addition of acid could cause some degree of depolymerization during precipitation,^[50]^ and these fragments were further removed during centrifugation and dialysis. In the case of PB and BB lignins (Figure 5b, c), the small‐molecular‐weight fragments were not recovered by acid or 70 % ethanol precipitation of lignin, which could be due to the increased solubility of such small fragments that did not precipitate from the solution during the LNP preparation, and they did not sediment during centrifugation step or leaked out through the dialysis membranes. Overall, among the three methods of LNP preparation, the acetone/water (3 : 1) method gave the most similar molar mass distribution and chromatograms as the starting lignin.

### Phenolic content evaluation

**Figure 6:**
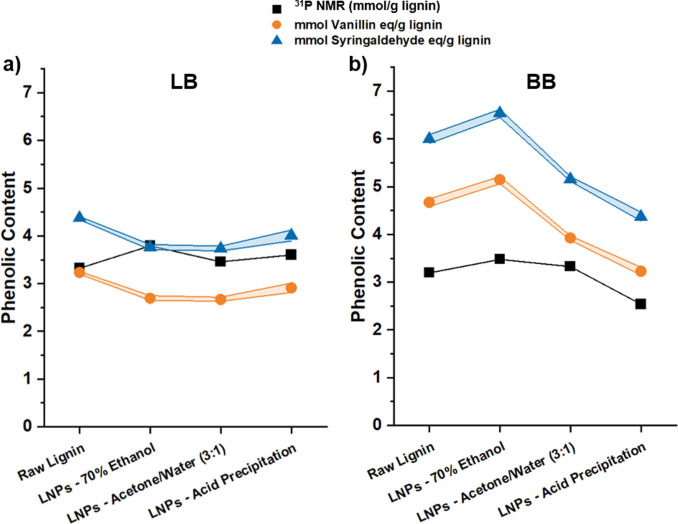
Determination of the phenolic content in (a) LB and (b) BB raw lignins and respective LNPs prepared with the 3 different approaches, using conventional ^31^P NMR spectroscopy and also UV/Vis spectroscopy (Folin‐Ciocalteu reagent) with vanillin or syringaldehyde as standards (n=3).

The lignin fractionation driven by the different solvent/anti‐solvent approaches to prepare LNPs could also affect the phenolic content of the resulting LNPs. In order to find a simple and fast method to determine the phenolic content of the softwood and hardwood lignin samples, we compared the values obtained using the conventional and established ^31^P NMR spectroscopy with two simple and convenient UV/Vis spectrometry approaches (Figure 6).

In the UV/Vis spectrometry, the Folin‐Ciocalteu reagent reacts with phenolic groups on the lignin structure, giving to the products a characteristic peak at 760 nm in the UV/Vis spectrum that is proportional to the content of phenolic hydroxy groups.^[51]^ Here, we used vanillin and syringaldehyde as standards, as they resemble the guaiacyl and syringyl units in the lignin structure, respectively. In general, the values obtained with the UV/Vis spectrometry approaches followed similar trends to the values measured with ^31^P NMR spectroscopy. However, the UV/Vis approach using syringaldehyde as a standard gave slightly different values compared to the vanillin standard, mainly due to the different molecular weight of the two compounds.

Regarding the sample preparation for each method, the conventional ^31^P NMR spectroscopy is a more sensitive technique, and it involves the dissolution of high amount of starting lignin and respective LNP powders; while in the UV/Vis method, a very small amount of raw lignins were dissolved in alkali aqueous solution, and the LNPs aqueous dispersions were diluted in water prior to the addition of Folin‐Ciocalteu reagent and the alkali solution. The Folin‐Ciocalteu reagent used in the UV/Vis approach does not react only with phenolic groups, but it can interact with any reducing substance present in the lignin structure, measuring the total reducing capacity of the lignin sample, which consequently leads to an overestimation of the phenolic content.[^52^, ^53^] Nevertheless, the Folin‐Ciocalteu method represents a simple, reproducible, and low‐cost approach to quantify the phenolic content when similar types of samples are compared, being useful when different oxidative treatments are applied to the same type of LNPs, for example.

### Effect of the ionic strength and pH on the stability of LNPs

**Figure 7:**
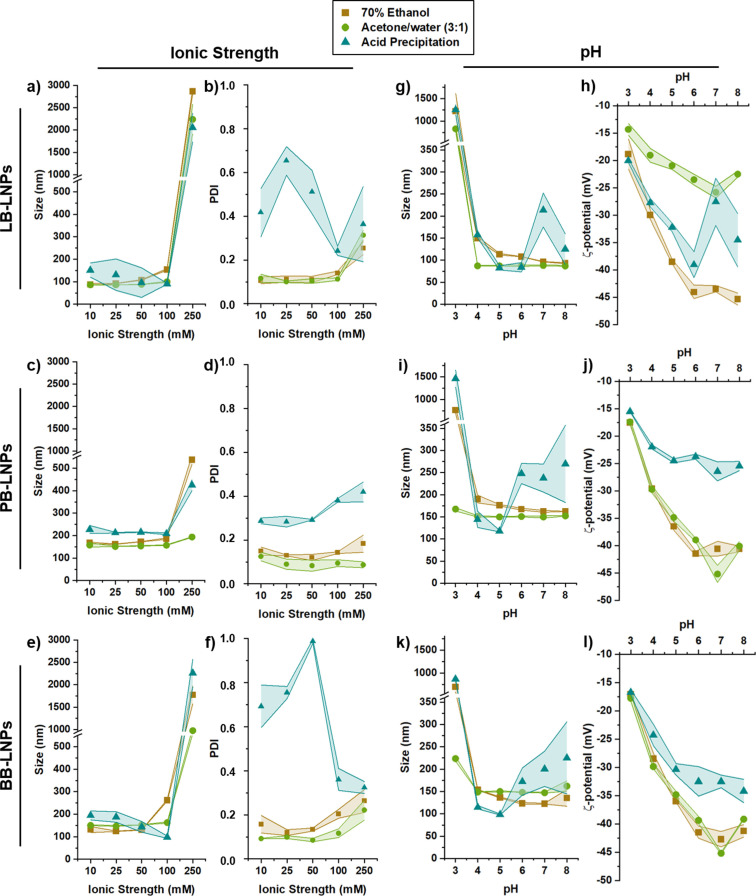
Effect of the ionic strength on the stability of (a,b) LB‐LNPs, (c, d) PB‐LNPs, and (e, f) BB‐LNPs, after incubation with 10–250 mm citric acid buffer pH 7 for 4 h, at room temperature. Effect of the pH on the stability of (g, h) LB‐LNPs, (I, j) PB‐LNPs, and (k, l) BB‐LNPs, after incubation with 25 mm citric acid buffer pH 3–8 for 4 h, at room temperature. Lines represent standard deviation values (n=3).

The stability of LNPs over time in media with different properties is an important factor that can determine the final application of the nanoparticles. Therefore, we evaluated the influence of the ionic strength and pH of citric acid buffers on the stability of LNPs, in terms of size, PDI, and ζ‐potential measured by DLS (Figure 7).

In order to evaluate the effect of the ionic strength on the LNP stability, the LNPs were incubated with different citric acid buffer solutions at pH 7 with ionic strength ranging between 10 and 250 mm (Figure 7a–f). In general, the hydrodynamic diameters of the three types of LNPs prepared with 70 % ethanol and acetone/water (3 : 1) methods remained similar as the ionic strength increased up to 100 mm, being approximately 100, 160, and 150 nm for LB‐, PB‐, and BB‐LNPs, respectively. At these ionic strengths, the citrate seems to stabilize the nanoparticles in suspension. However, the size of these LNPs increased significantly to over than 1000 nm for LB‐ and BB‐LNPs, when the ionic strength of the citric acid buffer was 250 mm, along with an increase in the PDI values. These results suggested an aggregation behavior mainly due to the interaction of citrate ions with the nanoparticles’ surface. Moreover, the LNPs prepared with the acid precipitation approach presented fluctuations in the PDI values, which can be due to the initial morphology of these particles that are not individual spherical nanoparticles, but aggregates or clusters of smaller particles.

The effect of the pH of citric acid buffer on the LNP stability was also assessed after incubating the LNPs with 25 mm citric acid buffer solutions at pH ranging from 3 to 8 (Figure 7g–l). Between pH 4 and 8, the size of LNPs prepared with 70 % ethanol and acetone/water (3 : 1) methods remained stable, but the LNP size dramatically increased at pH 3 to over 900 nm. This aggregation behavior started to occur when the pH gets closer to the isoelectric point and the carboxy groups become protonated, which induced the intermolecular hydrogen bonding between particles.[^13^, ^54^] Interestingly, the size of PB‐ and BB‐LNPs prepared with the acetone/water (3 : 1) approach did not experience a substantial increase at this pH, suggesting that these LNPs are stable at a wider pH range, which can increase the application of such LNPs. As indicated by the ζ‐potential values, the surface charge of the LNPs also increased as the pH decreased, as a consequence of the protonation of the carboxylic groups. The LNPs are highly negatively charged at neutral pH due to the hydroxy and carboxy groups on their surfaces, which stabilizes the LNP dispersion due to the electrostatic repulsion. Regarding the LNPs prepared with the acid precipitation method, the size and ζ‐potential of LNPs did not follow exactly the same trend, which can be ascribed again to the morphology of these LNPs.

Overall, the produced LNPs are stable at low to moderate ionic strength and over a wide range of pH, in particular the LNPs prepared with the acetone/water (3 : 1) method.

## Conclusion

The physicochemical properties of technical lignins can vary with their source (i. e., softwood, hardwood, or grass), which will influence the composition of lignins in terms of the proportion of structural units. In this study, we evaluated the effect of the solvent fractionation of lignin in the fabrication of lignin nanoparticles (LNPs) using three eco‐friendly approaches, and the LNPs were systematically characterized. The lignin fractionation due to the use of different solvents to prepare LNPs led to a different lignin dissolution efficacy, driven by the different phenolic hydroxy content and molar mass of technical lignins, which ultimately affected the LNP yield. The average size of three types of LNPs was found to be inversely proportional to the molar mass of the technical lignins [kraft LignoBoost (softwood, LB) >BLN birch lignin (hardwood, BB) >alkali Protobind 1000 (grass, PB)]. Furthermore, all methods allowed the preparation of homogeneous and monodispersed LNPs, in particular the acetone/water (3 : 1), with a negative surface charge. Among the three methods to obtain LNPs, the acetone method gave the most similar molar mass distribution as the starting lignin.

Overall, our results revealed that the acetone/water (3 : 1) mixture was the simplest and the most effective solvent tested to obtain homogenous, small, and spherical LNPs from the three technical lignins, with the highest LNP yields. This method also allowed the recovery of the organic solvent by rotary evaporation. Additionally, the resulting LNPs showed an improved stability at different ionic strengths and a wider pH range compared to the other preparation methods, which can greatly increase their application in many fields, such as pharmaceutical and food sciences.

## Experimental Section

### Materials

Three different lignin samples were selected for this study. Softwood kraft Lignoboost was provided by Stora Enso (Finland). Hardwood birch lignin (*Betula L*.) was isolated using the BLN process, and obtained from CH Bioforce Oy (Espoo, Finland). Protobind 1000 was extracted from wheat straw by the soda process and acquired from GreenValue SA (Switzerland). Ethanol (Etax Aa, ≥99.5 %) was purchased from Altia Oy (Finland). Acetone for HPLC (≥99.9 %), acetyl bromide, vanillin, syringaldehyde, and citric acid monohydrate were acquired from Sigma‐Aldrich (Finland). Acetic acid (glacial) 100 % for analysis EMPARTA® ACS, Folin‐Ciocalteu reagent, and sodium carbonate were purchased from Merck (Finland). NMR solvent DMSO‐d6 was acquired from Eurisotop (Saint‐Aubin, France).

### Fabrication methods of LNPs

The ethanol nanoprecipitation method we used to prepare LNPs was adapted from the approach previously reported by Sipponen et al.^[13]^ Briefly, the technical lignins were dissolved with a 70 % (*v*/*v*) aqueous ethanol solution at initial concentration of 10 mg mL^−1^ and stirred overnight. Afterwards, 50 mL lignin solution was filtered using hydrophilic polypropylene membrane filters with a 0.45 μm pore size (Whatman) to remove undissolved solids, and 190 mL of MilliQ‐water was rapidly poured into the magnetically stirred lignin solution, so that the final percentage of ethanol dropped to 25 %. After overnight stirring, the LNPs were collected and centrifuged for 20 min at 100000 *g*, resuspended with MilliQ‐water, and redispersed using ultrasonication (Branson digital sonicator) at a frequency of 20 kHz, 50 % oscillation amplitude (100 W) for 60 s. The acetone nanoprecipitation approach for the fabrication of LNPs was adapted from Farooq et al.,^[26]^ in which 2 g of technical lignins was dissolved in 200 mL of acetone/water 3 : 1 (*v*/*v*) mixture and stirred for 3 h, followed by filtration using a glass microfiber filter (Whatman GF/F, pore size 0.7 μm). The obtained solution was rapidly poured into 400 mL of MilliQ‐water under vigorous stirring. Acetone was further removed by evaporation under reduced pressure at 40 °C to obtain the LNPs dispersions. The acid precipitation method to prepare LNPs was combined with mild ultrasonication, as previously reported by Agustin et al.^[18]^ Briefly, 5 g of technical lignins was dissolved in 100 mL of 0.25 m NaOH solution and stirred vigorously, followed by rapid addition of 125 mL of 0.25 m HCl to lower the pH to about 1. The resulting mixture was then centrifuged for 7 min at 14000 *g* to remove most of the salts and acids, and the pellet was further collected, diluted with water, and then dialyzed against distilled water, using Spectra/Por 1 (6–8 kDa molecular weight cutoff) for 3 days, replacing the water periodically. Finally, the dialyzed mixture was kept in an ice bath and sonicated using a Branson digital sonicator at a frequency of 20 kHz and 80 % amplitude (100 W) for a total of 5 min.

### Dynamic light scattering

The average hydrodynamic diameter, polydispersity index, and ζ‐potential of LNPs was measured by DLS, using a Malvern Zetasizer Nano ZS instrument (Malvern Instruments Ltd, UK). For that, the samples were diluted in MilliQ‐water at a concentration of 500 μg mL^−1^.

### Transmission electron microscopy

To confirm the size distribution and morphology of LNPs, the particles were visualized by TEM (Jeol JEM‐1400, Jeol Ltd., Tokyo, Japan), using an acceleration voltage of 80 kV. For the sample preparation, a droplet of LNPs dispersion was placed on a carbon‐coated copper grid, blotted using a filter paper, and then air‐dried before analysis.

### Fourier‐transform infrared spectroscopy

The FTIR spectra of the technical lignins and LNPs were recorded using SpectrumOne (PerkinElmer, Turku, Finland), equipped with a universal attenuated total reflectance accessory. The FTIR spectra were recorded at room temperature between 4000–600 cm^−1^ with a resolution of 4 cm^−1^ and number of 64 scans, and the baseline was corrected using the built‐in software.

### Small‐angle X‐ray scattering analysis

SAXS was measured using a Xenocs Xeuss 3.0 C device equipped with a GeniX 3D Cu microfocus source (*λ*=1.542 Å) and EIGER2 R 1 M hybrid pixel detector at a sample‐to‐detector distance of 109 cm. Sample solutions at a concentration of 4 mg mL^−1^ [70 % ethanol and acetone/water (3 : 1) LNPs] or 20 mg mL^−1^ (acid precipitation LNPs) were injected in a capillary flow cell, and data was collected for each sample at the same spot on the glass capillary. The measured intensities were corrected for cosmic radiation, integrated azimuthally over a full circle, divided by transmitted beam intensity, normalized to absolute scale using a glassy carbon sample, and background‐subtracted using data measured for pure water. The corrected and background‐subtracted intensities were finally scaled to units of mm^−1^ by dividing them by the thickness of the capillary (1.5 mm). The magnitude of the scattering vector corresponding to scattering angle 2*θ* was defined as *q=*4*π*sin(*θ*)/*λ*.

### Nuclear magnetic resonance spectroscopy

For both ^1^H NMR and 2D HSQC analysis, 30 mg of lignin/LNP samples was dissolved in 0.7 mL of DMSO‐d6, and the analysis was performed at 27 °C. The spectra were acquired using a Bruker Avance 850 MHz III high‐definition spectrometer equipped with a cryoprobe (Bruker Corp., MA). The pulse width for all samples and experiments was 7.98 μs. The ^1^H NMR experiments were obtained using the zg pulse program and the following parameters: D1 delay 10 s, number of dummy scans 8, and number of scans 32. The 2D HSQC NMR experiments were performed using the pulse program hsqcedetgpsisp.2 and the following parameters: the size of the FID 2048, D1 delay 2 s, number of dummy scans 32, and number of scans 16. The spectral widths used were 12 ppm in the ^1^H dimension and 220 ppm in the ^13^C dimension.

For the ^31^P NMR spectroscopy, 30 mg of each sample was dissolved in 400 μL of a 1 : 1.6 mixture of CDCl_3_/pyridine after which 100 μL of an internal standard solution (e‐HNDI, *endo*‐*N*‐hydroxy‐5‐norbornene‐2,3‐dicarboximide, 0.12 m in CDCl_3_/pyridine 1 : 1.6) was added, followed by 50 μL of a relaxation agent [Cr(acac)3, 11.4 mg mL^−1^ in CDCl_3_/pyridine 1 : 1.6]. Finally, 100 μL of a phosphitylation reagent Cl‐TMDP (2‐chloro‐4,4,5,5‐tetramethyl‐1,3,2‐dioxaphospholane) was added and the solution was stirred for 1 h to ensure complete derivatization before transferring the samples to NMR tubes. The spectra were acquired on a Bruker AVANCE III spectrometer operating at 500.10 MHz (1H) and 202.44 MHz (31P), equipped with a BB/1H SmartProbe. The data was acquired using a standard pulse program for quantitative ^31^P NMR spectroscopy, using inverse‐gated decoupling (zgig). The relaxation delay was set to 10 s, and the acquisition time to 3.3 s resulting in a total repetition time of 13.3 s to ensure complete relaxation between scans. A total of 64 scans were acquired on each sample. Spectra were recorded from 117 to 166 ppm to place the region of interest (approximately 130–154 ppm) in the center of the spectrum.

### Size exclusion chromatography

The molar mass distribution of the technical lignins and respective LNPs prepared with the different methods was analyzed following the acetobromination procedure reported by Asikkala et al.^[55]^ Briefly, 2.3 mL of glacial acetic acid was added to 10 mg of dried lignin sample in a reaction flask, the mixture was stirred for 15 min, followed by dropwise addition of 0.25 mL acetyl bromide. The reaction mixture was stirred for 1 h at room temperature and rotary evaporated to remove the excess acetic acid and acetyl bromide. The final product was further dried in a vacuum, at 30 °C for 45 min, before chromatographic analysis. SEC was performed using the Waters 515 HPLC pump, Biotech DEGASi GPC Degasser and Waters 717 plus autosampler. The separation was performed at 30 °C using Waters styragel HR 1, 2, and 4 columns, with THF as eluent at a flow rate of 0.80 mL min^−1^. Detection was done using Waters 2487 Dual λ Absorbance Detector and Waters 2410 Differential Refractometer. Polystyrene standards provided by Polymer Standards Service were used as reference and Omnisec 4.7 software was used for data evaluation.

### Phenolic content evaluation

The total amount of phenolic hydroxy groups was also estimated with a spectrophotometric method based on the Folin‐Ciocalteu reagent.^[56]^ For that, 50 μL of aqueous dispersions of LNP or alkali‐solubilized technical lignins (0.5 mg mL^−1^) was diluted with 1.8 mL of MilliQ‐water, and further mixed with 150 μL Folin‐Ciocalteu reagent. After around 6 min, 500 μL of sodium carbonate solution (20 %, *w*/*w*) was added, and the resulting mixtures were mixed and kept at 40 °C for 30 min. Finally, the absorbance at 760 nm of the blue‐colored samples was measured using a UV/Vis spectrophotometer (UV‐1800 Shimadzu) with the UV probe 2.70 software. The amount of free phenolic groups was quantified from standard curves based on vanillin (4‐hydroxy‐3‐methoxybenzaldehyde) or syringaldehyde (4‐hydroxy‐3,5‐dimethoxybenzaldehyde), which represent the guaiacyl and syringyl unit commonly found in wood lignins.

### Stability of LNPs at different ionic strength and pH

The stability of the LNPs was evaluated by following the changes in their average size, PDI, and ζ‐potential using DLS. For that, 0.5 mg mL^−1^ of LNP suspensions was incubated for 4 h at room temperature in 10–250 mm citric acid buffer pH 7 or 25 mm citric acid buffer pH 3–8 (adjusted with 10 m NaOH) in order to study the effect of ionic strength and pH variation in the LNPs stability. All the experiments were performed in triplicate.

## Conflict of interest

The authors declare no conflict of interest.

## Supporting information

As a service to our authors and readers, this journal provides supporting information supplied by the authors. Such materials are peer reviewed and may be re‐organized for online delivery, but are not copy‐edited or typeset. Technical support issues arising from supporting information (other than missing files) should be addressed to the authors.

Supporting Information
