# Supplementary material for: Green Fabrication Approaches of Lignin Nanoparticles from Different Technical Lignins: A Comparison Study
Source: ChemSusChem. 2021 Oct 4;14(21):4718–30. doi: 10.1002/cssc.202101356 (PMC8596756; doi:10.1002/cssc.202101356)
Supplement: Supplementary file 1 — Supporting Information [file CSSC-14-4718-s001.pdf]

ChemSusChem

Supporting Information

Green Fabrication Approaches of Lignin Nanoparticles from Different Technical Lignins: A Comparison Study

Patrícia Figueiredo,* Maarit H. Lahtinen, Melissa B. Agustin, Danila Morais de Carvalho, Sami-Pekka Hirvonen, Paavo A. Penttilä, and Kirsi S. Mikkonen*© 2021 The Authors. ChemSusChem published by Wiley-VCH GmbH. This is an open access article under the terms of the Creative Commons Attribution License, which permits use, distribution and reproduction in any medium, provided the original work is properly cited.

RESULTS AND DISCUSSION

Table S1. Molar mass distribution for the different types of lignin (LB, softwood; PB, wheat straw; BB, hardwood).

Sample	LB			PB			BB		
	M_n (Da)	M_w (Da)	M_w/M_n	M_n (Da)	M_w (Da)	M_w/M_n	M_n (Da)	M_w (Da)	M_w/M_n
**Raw Lignin**	1399	22282	15.9	806	12496	15.5	807	16766	20.8
**70% Ethanol**	1181	6025	5.1	957	14355	15.0	942	9403	10.0
**Acetone/Water (3:1)**	1451	20225	13.9	877	14174	16.2	831	13150	15.8
**Acid Precipitation**	1320	13457	10.2	952	12417	13.0	1006	14225	14.1

Abbreviations: M_n, number-average molecular weight; M_w, weight-average molecular weight.

Table S2. Content in hydroxyl and carboxyl groups of softwood LB and hardwood BB by ^{31}P nuclear magnetic resonance (^{31}P NMR), expressed in mmol/g.

Samples	**Aliphatic OH** (145–150 ppm)	**C5 substituted**(LB)/ **Syringyl**(BB) (140–144.5 ppm)	**Guaiacyl** (139–140 ppm)	***p*-Hydroxyphenyl** (137.8 ppm)	**Total Phenolic OH**	**S/G ratio**	**COOH** (133.6–136 ppm
**Raw LB**	2.19	1.50	1.72	0.10	3.32	0.87	0.32
**LB-LNPs** (70% Ethanol)	2.02	1.68	2.00	0.11	3.80	0.84	0.29
**LB-LNPs** (Acetone/water 3:1)	2.26	1.64	1.72	0.10	3.46	0.96	0.35
**LB-LNPs** (Acid precipitation)	1.72	1.57	1.92	0.12	3.60	0.82	0.47
**Raw BB**	1.04	2.59	0.61	0	3.20	4.27	0.56
**BB-LNPs** (70% Ethanol)	1.01	2.78	0.70	0	3.48	3.97	0.53
**BB-LNPs** (Acetone/water 3:1)	1.14	2.65	0.68	0	3.33	3.93	0.62
**BB-LNPs** (Acid precipitation)	1.43	2.07	0.47	0	2.54	4.42	0.64

Table S3. Results of fits of a sphere model with log-normal size distribution to the SAXS intensities.[1]

	**Diameter, mean (nm)**	**Diameter, median (nm)**	**Diameter, mode (nm)**	**Standard deviation (nm)**
**LB-LNPs – 70% Ethanol** (4 mg/ml)	43.7	40.9	35.8	16.5
**LB-LNPs – Acetone/Water (3:1)** (4 mg/ml)	46.4	44.0	39.6	15.5
**PB-LNPs – 70% Ethanol** (4 mg/ml)	39.6	35.3	28.0	20.1
**PB-LNPs – Acetone/Water (3:1)** (4 mg/ml)	69.6	66.4	60.6	21.6
**BB-LNPs – 70% Ethanol** (4 mg/ml)	41.1	36.3	28.3	21.8
**BB-LNPs – Acetone/Water (3:1)** (4 mg/ml)	67.5	64.8	59.8	19.6

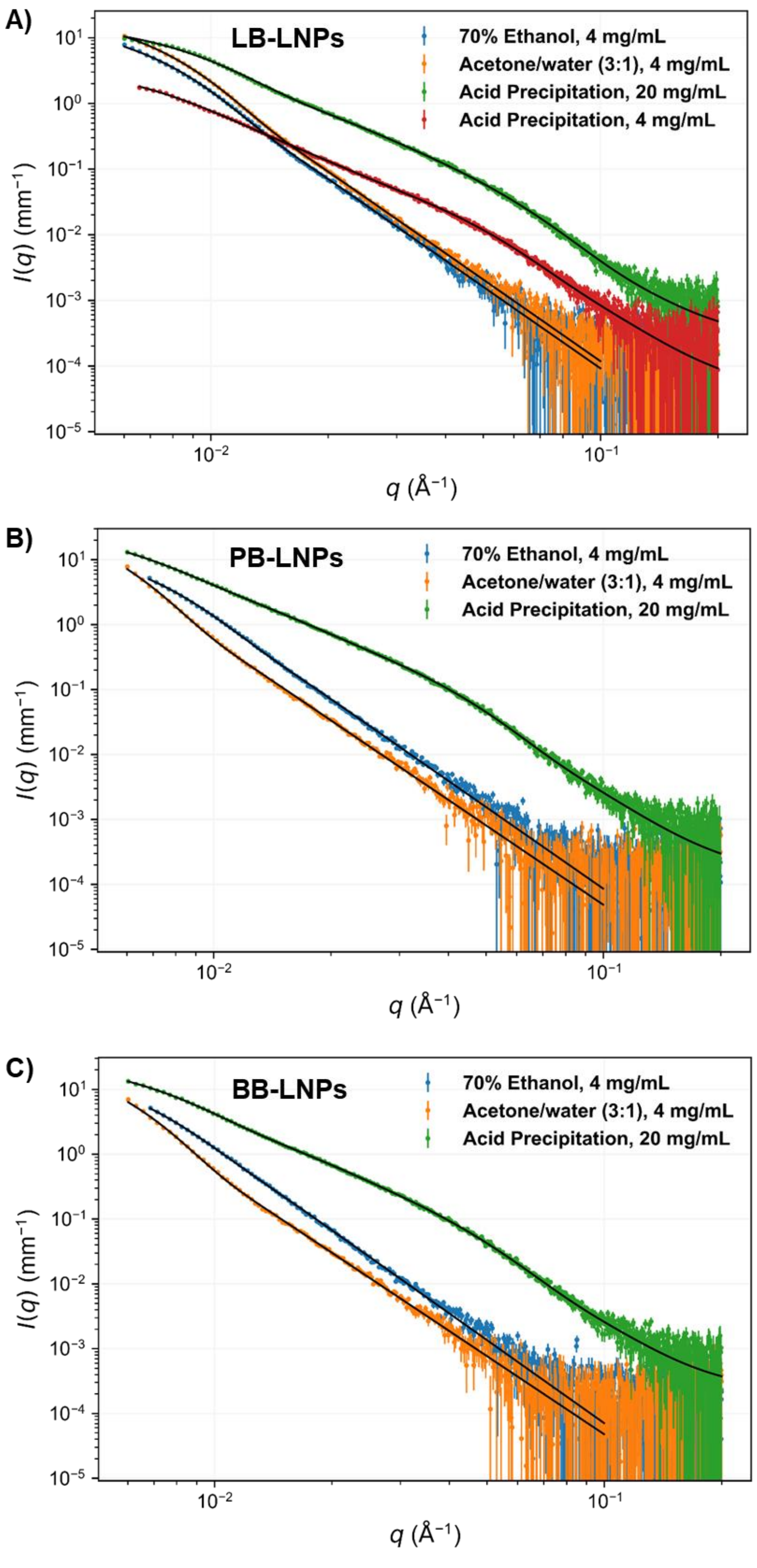

Figure S1. SAXS intensities (points) and fits (solid line) of the unified exponential/power-law model with one (Acetone/water 3:1 and 70% Ethanol methods) or two (Acid precipitation approach) levels of structural hierarchy: **A)** LB-LNPs; **B)** PB-LNPs, and **C)** BB-LNPs.

Table S4. Results of fits of the unified exponential/power-law model to the SAXS intensities.[2,3]

	**Radius of gyration, level 1 (nm)**	**Sphere diameter, level 1 (nm)**	**Radius of gyration, level 2 (nm)**	**Sphere diameter, level 1 (nm)**
**LB-LNPs – 70% Ethanol** (4 mg/ml)	29.4	75.9	-	-
**LB-LNPs – Acetone/Water (3:1)** (4 mg/ml)	29.3	75.8	-	-
**LB-LNPs – Acid Precipitation** (4 mg/ml)	25.4	65.7	4.6	11.8
**LB-LNPs – Acid Precipitation** (20 mg/ml)	22.1	57.0	3.4	8.9
**PB-LNPs – 70% Ethanol** (4 mg/ml)	31.0	80.0	-	-
**PB-LNPs – Acetone/Water (3:1)** (4 mg/ml)	43.7	112.9	-	-
**PB-LNPs – Acid Precipitation** (20 mg/ml)	31.0	80.1	5.3	13.7
**BB-LNPs – 70% Ethanol** (4 mg/ml)	34.6	89.4	-	-
**BB-LNPs – Acetone/Water (3:1)** (4 mg/ml)	40.9	105.6	-	-
**BB-LNPs – Acid Precipitation** (20 mg/ml)	28.0	72.2	4.8	12.3

Table S5. IR absorption bands and respective type of vibration for the main lignin functional groups.[4,5]

**IR band (cm^{-1})**	**Type of vibration**
3500–3100	Stretching vibrations of alcohol and phenol –OH groups involved in hydrogen bonds
2920–2850	Stretching vibrations of C–H bonds in methoxy group
1720	Stretching vibrations of C=O bonds at β location and in unconjugated –COOH group
1600	Stretching vibrations of C=O bonds at α- and γ-locations
1512	Aromatic ring vibrations
1465	
1427	
1330	Syringyl ring breathing and stretching vibrations of C–O bonds
1230	
1270	Vibrations of guaiacyl rings and stretching vibrations of C–O bonds
1150	Deformation vibrations of C–H bonds in guaiacyl rings
1130	Deformation vibrations of C–H bonds in syringyl rings
1085	Deformation vibrations of C–O bonds in secondary alcohols and aliphatic ethers
1030	Deformation vibrations of C–H bonds in the aromatic guaiacyl rings, and deformation vibrations of C–O bonds in primary alcohols
860-750	Deformation vibrations of C–H bonds in the aromatic rings (guaiacyl, syringyl)

Table S6. Assignment for the carbon-hydrogen correlation signals of lignin structures in BB, LB, and PB lignins.[3,6–9]

Structural units	δ_C (ppm)	δ_H (ppm)	Assignments	Identified in
Syringyl ($S_{2,6}$)	103.6	6.68	C_2/H_2 and C_6/H_6 in S-units	BB, PB
Guaiacyl (G_2)	110.9	7.14	C_2/H_2 in G-units	BB, LB, PB
Guaiacyl (G_5)	115.5	6.75	C_5/H_5 in G-units	BB, LB, PB
Guaiacyl (G_6)	119.5	6.93	C_6/H_6 in G-units	BB, LB, PB
p-hydroxyphenyl ($H_{2,6}$)	127.5	7.21	C_2/H_2 and C_6/H_6 in H-units	BB, LB, PB
β-aryl ether (β-*O*-4)	**71.0**	**4.75**	β-*O*-4 signal in α	LB, PB
β-aryl ether (β-*O*-4)	84.2	4.26	β-*O*-4 signal in β	LB, PB
β-aryl ether (β-*O*-4)	59.9	3.24-3.60	β-*O*-4 signal in γ	LB, PB
Phenylcoumaran (β-5)	**86.8**	**5.45**	β-5 signal in α	LB, PB
Phenylcoumaran (β-5)	53.4	3.45	β-5 signal in β	LB, PB
Phenylcoumaran (β-5)	62.9	3.71	β-5 signal in γ	LB, PB, BB
Dibenzodioxocin (5-5)	82.9	4.81	5-5/β-*O*-4 signal in α	BB, PB
Resinol (β-β)	**85.1**	**4.61**	β-β signal in α	LB, PB, BB
Resinol (β-β)	53.6	3.04	β-β signal in β	LB, PB, BB
Resinol (β-β)	71.0	4.14; 3.77	β-β signal in γ	LB, PB, BB
Secoisolariciresinol	33.8	2.53/2.48	Secoisolariciresinol in α	LB, PB
Secoisolariciresinol	**42.4**	**1.88**	Secoisolariciresinol in β	LB, PB
Methoxyl group	55.5	3.75-3.82	C/H in -OMe in G- and S-units	LB, PB, BB

Table S7. Assignment for the relevant carbon-hydrogen correlation signals of polysaccharides linkages identified in BB, LB, and PB lignins.[9,10]

Structural units	δ_C (ppm)	δ_H (ppm)	Assignments[a]	Identified in
Ara_1	107.8	4.76	C_1/H_1 in β-L-arabinopyranoside	LB
Ara_5	62.3	3.42/3.36	C_5/H_5 in β-L-arabinopyranoside	BB, LB
Glc_1	102.0	4.32	C_1/H_1 in β-D-glucopyranoside	BB, LB, PB
Glc_6/Man_6	60.0	3.59	C_6/H_6 in β-D-gluco-/mannopyranoside	LB, PB
Xyl_1	101.5	4.38	C_1/H_1 in β-D-xylopyranoside	LB, BB, PB
Xyl_2	72.7	3.03	C_2/H_2 in β-D-xylopyranoside	BB, LB, PB
$Xyl_2(r_\alpha)$	69.7	3.27	C_2/H_2 in β-D-xylopyranoside of xylans reduction end	BB, PB
Xyl_2-Ac	73.0	4.40	C_2/H_2 in 2-*O*-acetyl-β-D-xylopyranoside	BB, PB
Xyl_3	74.5	3.28	C_3/H_3 in β-D-xylopyranoside	BB, LB, PB
Xyl_4	75.7	3.51	C_4/H_4 in β-D-xylopyranoside	BB, LB, PB
Xyl_5	62.8	3.78/3.17	C_5/H_5 in β-D-xylopyranoside	BB, LB, PB
Xyl_4(nr)	66.0	3.31	C_4/H_4 in β-D-xylopyranoside of xylans non-reducing end	LB, PB
Man_3	76.6	3.08	C_3/H_3 in β-D- mannopyranoside	BB, PB
Man_3-Ac	73.0	4.82	C_3/H_3 in 3-*O*-acetyl-β-D- mannopyranoside	BB
Man_4	79.3	3.38	C_4/H_4 in β-D- mannopyranoside	BB
Gal_1	105.0	4.26	C_1/H_1 in β-D-galactopyranoside	LB

References

[1] M. H. Sipponen, A. Henn, P. Penttilä, M. Österberg, *Chem. Eng. J.* **2020**, *393*, 124711.

[2] G. Beaucage, *J. Appl. Crystallogr.* **1995**, *28*, 717.

[3] M. B. Agustin, P. A. Penttilä, M. Lahtinen, K. S. Mikkonen, *ACS Sustain. Chem. Eng.* **2019**, *7*, 19925.

[4] B. Ahvazi, É. Cloutier, O. Wojciechowicz, T. D. Ngo, *ACS Sustain. Chem. Eng.* **2016**, *4*, 5090.

[5] P. Figueiredo, C. Ferro, M. Kemell, Z. Liu, A. Kiriazis, K. Lintinen, H. F. Florindo, J. Yli-Kauhaluoma, J. Hirvonen, M. A. Kostiainen, H. A. Santos, *Nanomedicine* **2017**, *12,* DOI 10.2217/nnm-2017-0219.

[6] T. M. Liitiä, S. L. Maunu, B. Hortling, M. Toikka, I. Kilpeläinen, *J. Agric. Food Chem.* **2003**, *51*, 2136.

[7] C. S. Lancefield, H. J. Wienk, R. Boelens, B. M. Weckhuysen, P. C. A. Bruijnincx, *Chem. Sci.* **2018**, *9*, 6348.

[8] L. Lagerquist, A. Pranovich, A. Smeds, S. von Schoultz, L. Vähäsalo, J. Rahkila, I. Kilpeläinen, T. Tamminen, S. Willför, P. Eklund, *Ind. Crops Prod.* **2018**, *111*, 306.

[9] J. Rencoret, A. Gutiérrez, L. Nieto, J. Jiménez-Barbero, C. B. Faulds, H. Kim, J. Ralph, Á. T. Martínez, J. C. del Río, *Plant Physiol.* **2011**, *155*, 667.

[10] N. Giummarella, M. Lawoko, *ACS Sustain. Chem. Eng.* **2017**, *5*, 5156.
